# Bostrycin inhibits proliferation of human lung carcinoma A549 cells via downregulation of the PI3K/Akt pathway

**DOI:** 10.1186/1756-9966-30-17

**Published:** 2011-02-08

**Authors:** Wei-Sheng Chen, Jun-Na Hou, Yu-Biao Guo, Hui-Ling Yang, Can-Mao Xie, Yong-Cheng Lin, Zhi-Gang She

**Affiliations:** 1 Department of Pulmonary and Critical Care Medicine, the First Affiliated Hospital of Sun Yat-Sen University, Guangzhou 510080, China; 2 Department of Physiopathology, Zhongshan School of Medicine, Sun Yat-Sen University, Guangzhou 510080, China; 3 Marine Microorganism Lab, Institute of Chemistry and Chemical Engineering, Sun Yat-Sen University, Guangzhou 510080, China

## Abstract

**Background:**

Bostrycin is a novel compound isolated from marine fungi that inhibits proliferation of many cancer cells. However, the inhibitory effect of bostrycin on lung cancers has not been reported. This study is to investigate the inhibitory effects and mechanism of bostrycin on human lung cancer cells in vitro.

**Methods:**

We used MTT assay, flow cytometry, microarray, real time PCR, and Western blotting to detect the effect of bostrycin on A549 human pulmonary adenocarcinoma cells.

**Results:**

We showed a significant inhibition of cell proliferation and induction of apoptosis in bostrycin-treated lung adenocarcinoma cells. Bostrycin treatment caused cell cycle arrest in the G0/G1 phase. We also found the upregulation of microRNA-638 and microRNA-923 in bostrycin-treated cells. further, we found the downregulation of p110α and p-Akt/PKB proteins and increased activity of p27 protein after bostrycin treatment in A549 cells.

**Conclusions:**

Our study indicated that bostrycin had a significant inhibitory effect on proliferation of A549 cells. It is possible that upregulation of microRNA-638 and microRNA-923 and downregulaton of the PI3K/AKT pathway proteins played a role in induction of cell cycle arrest and apoptosis in bostrycin-treated cells.

## Background

Lung cancer is the most common type of cancer worldwide. Despite recent advances in surgical techniques and chemotherapy/radiotherapy strategies, the long-term survival rates remain poor. There is therefore an urgent need to develop new therapeutic strategies in order to significantly improve the prognosis in lung cancer patients. Growth factor signaling pathways have been shown to be important targets in lung cancer therapy. Targeting such intracellular pathways that regulate proliferation, apoptosis, metastasis and resistance to chemotherapy represents an important therapeutic strategy for lung cancer [1].

Marine microorganisms can grow under adverse conditions such as low temperatures, high pressures, and poor nutrition. The diversity of biological activities in these environments exceeds those of land organisms. Some metabolites from these marine microorganisms have novel structures and biological activities including anticancer, antiviral and immune enhancement properties. A recent study on marine pharmacology coordinated by multiple countries demonstrated antitumor activity in a number of natural products derived from marine invertebrates, algae, fungi, and bacteria, although the mechanisms of action are still unknown [2].

Bostrycin, a novel compound isolated from marine fungi in South China Sea, has been shown to inhibit cell growth in in prostate cancer and gastric cancer [3,4]. However, since the antitumor effect of bostrycin in lung cancer is not known, we explored the effect of bostrycin treatment in lung cancer cells and investigated the mechanisms underlying the inhibitory effect of bostrycin in lung cancers.

## Materials and methods

### Cell line and cell culture

The human pulmonary adenocarcinoma cell line A549 was obtained from the Cell Bank of the Animal Experiment Center, North School Region, Sun Yat-Sen University. Cells were cultured in DMEM medium (low glucose) supplemented with 10% newborn calf serum at 37°C with 5% CO_2_. Cells were digested with 0.25% trypsin and subcultured at 70% to 80% confluence Exponentially growing A549 cells were used for all assays.

### Test compound

Bostrycin (hydroxy-methoxy-tetrahydro-5-methyl anthracene dione), a novel compound isolated from marine fungi in P.R. China, was supplied by Marine Microorganism Laboratory, Institute of Chemistry and Chemical Engineering, Sun Yat-Sen University. The chemical structure of bostrycin is shown inAdditional file 1, Figure S1.

### Major reagents

Newborn calf serum, DMEM (low glucose), 0.25% trypsin digest, and Trizol reagent were purchased from GIBCO (Invitrogen Corporation, Carlsbad, CA, USA). MTT and DMSO were obtained from Sigma Corporation. Mouse anti-human phospho-Akt monoclonal antibody (mAb), rabbit anti-human p110α mAb, rabbit anti-human p27 mAb, horseradish peroxidase (HRP)-conjugated goat anti-mouse IgG (secondary antibody), HRP-conjugated goat anti-rabbit IgG (secondary antibody), and prestained protein molecular weight marker were purchased from Cell Signaling Technology (USA).

### Measurement of cell growth inhibition by MTT assay

A549 cells were seeded in 96-well plates (5 × 10^3 ^cells per well) and treated with bostrycin (10, 20, and 30 μmol/L). Negative control wells (containing cells but not bostrycin), and the blank control (only medium) were plated with 6 replicates each. Untreated and treated cells were cultured at 37°C with 5% CO_2 _for 12 hours. MTT solution (20 μL) was added to each well and mixed; the wells were then incubated for an additional 4 hours. Culture supernatant was removed, DMSO (150 μL) was added to each well and vortexed at low speed for 10 minutes to fully dissolve the blue crystals. Absorbance was measured at 570 nm (A_570_) and the percentage of growth inhibition of A549 cells was calculated at each time point and for each concentration of bostrycin according to the following formulae: % cell survival = (A570bostrycin group - A570blank)/(A570negative - A570blank) × 100% and % cell growth inhibition = 1 - % cell survival. Half maximal inhibitory concentration (IC50) values at respective times were then calculated using linear regression.

### Cell cycle and apoptosis rate assayed by flow cytometry

A549 cells were cultured in 6-well plates (1.5 × 10^5 ^cells per well) and treated with different concentrations (5, 10, and 20 μmol/L) of bostrycin or complete DMEM medium (for the control group) and incubated for 24, 48 or 72 hours. Culture supernatant from each group was pooled and the cells were fixed for 12 h with 1 ml of 75% ethanol (10^6 ^cells/ml) and transferred to 2 mL Eppendorf tubes for flow cytometry and propidium iodide (PI) staining. For PI staining, the cells were washed twice with cold PBS and centrifuged at 1000 g for 5 min. The pellet was washed twice in cold 0.1% Triton X-100 PBS and incubated at room temperature for 30 minutes with 300 μL DNA dye (containing 100 μg/mL propidium iodide and 20 U/mL RNase; Sigma Corporation). Flow cytometry analysis (BECKMAN-COULTER Co., USA) was performed. The cells were collected for the calculation of DNA amount for cell cycling analysis using a MULTYCYCLE software (PHEONIX, Co. USA). The extent of apoptosis was analyzed and quantified using WinMDI version 2.9 (Scripps Research Institute, La Jolla, CA, USA).

### Differential expression of microRNAs

#### Preparation of total RNA sample

A549 cells were cultured in 6-well plates (1.5 × 10^5 ^cells per well) and treated for 72 h with 10 μmol/L bostrycin for the bostrycin group or with complete medium for the control group. The cells were lysed in 1.5 mL of Trizol reagent and total RNA was prepared according to the manufacturer's instructions.

#### Microarray

Microarray analysis was performed using a service provider (LC Sciences, USA). The assay used 2-5 μg total RNA, which was size-fractionated using a YM-100 Microcon centrifugal filter (SIGMA). The small RNAs (<300 nucleotides) isolated were 3' extended using poly(A) polymerase. An oligonucleotide tag was then ligated to the poly(A) tail for fluorescent dye staining. Two different tags were used for the two RNA samples in dual-sample experiments. Hybridizations were performed overnight on a μParaflo microfluidic chip using a microcirculation pump (Atactic Technologies, Houston, TX, USA). Each detection probe on the microfluidic chip consisted of a chemically modified nucleotide-coding segment complementary to a target microRNA (miRBase; http://microrna.sanger.ac.uk/sequences/) or other RNA (control or customer-defined sequences). The probe also contained a spacer segment of polyethylene glycol to separate the coding segment from the substrate. The detection probes were made by *in situ *synthesis using PGR (photogenerated reagent chemistry). The hybridization melting temperatures were balanced by chemical modifications of the detection probes. Hybridization was done in 100 μL 6 × saline-sodium phosphate-EDTA buffer (0.90 M NaCl, 60 mMNa_2_HPO_4_, and 6 mM EDTA, pH 6.8) containing 25% formamide at 34°C and fluorescence labeling with tag-specific Cy3 and Cy5 dyes was used for detection. Hybridization images were collected using a laser scanner (GenePix 4000B, Molecular Device) and digitized using Array-Pro image analysis software (Media Cybernetics). Data were analyzed by first subtracting the background and then normalizing the signals using a LOWESS filter (locally weighted regression). For two-color experiments, the ratio of the two sets of detected signals (log 2 transformed; balanced) and *P *values of the *t *test were calculated. Differentially detected signals were those with *P *< 0.01.

#### RT-PCR

RT-PCR was performed using the TaqMan MicroRNA Reverse Transcription Kit (LC Sciences, USA) and the ABI PRISM 7000 Sequence Detection System (Life Technologies Corporation, Carlsbad, CA, USA). 2 μg RNA was used to synthesize single stranded cDNA according to the manufacturer's instructions. Real time PCR was performed to amplify the cDNA with the TaqMan Universal PCR Master Mix (LC Sciences, USA) as follows: amplification for 30 cycles at 94°*C *for 0.5 min, annealing at 55°*C *for 0.5 min, and extension at 72°*C *for 0.5 min; and then terminal elongation step at 72°*C *for 10 min and a final holding stage at 4°*C*. The amplification plots were viewed and the baseline and threshold values (as indicated in the instrument user guide) were set to analyze the results. The relative miRNA expression was calculated using 2^-ΔΔCt ^where ΔCt is the difference between target miRNA or reference miRNA Ct values in the treated and control samples. ΔΔCt is the difference between the above two ΔCt from target miRNA and reference miRNA.

### Western blotting

A549 cells (cultured in 6-well plate at 1.5 × 10^5 ^cells per well) were treated with 10 μmol/L bostrycin for 12, 24, 48, and 72 hours, and total proteins were extracted. Protein samples were separated by SDS-PAGE and electrophoretically transferred onto a polyvinylidene difluoride membrane (Millipore, USA). The membrane was blocked overnight at 4 degree in TBS-Tween 20 (TBST) buffer containing 5% skimmed milk powder. The membrane was washed with TBST (3 × 8 minutes). Membranes were then incubated overnight at 4°C in primary antibody (125 μL/cm^3^; diluted 1:1,000) with gentle shaking. The membranes were washed with TBST (3 × 8 minutes) and incubated for 1 h at room temperature in HRP-conjugated secondary antibody (125 μL/cm^3^; diluted 1:2,500). The membranes were washed with TBST (3 × 8 minutes) and protein signals were detected by chemiluminescence kit (Cell signaling Technology, USA).

### Statistical analysis

Normally distributed continuous variables were compared by one-way analysis of variance (ANOVA). When a significant difference between groups was apparent, multiple comparisons of means were performed using the Bonferroni procedure with type-I error adjustment. Data are presented as means ± SD. All statistical assessments were two-sided and evaluated at the 0.05 level of significant difference. Statistical analyses were performed using SPSS 13.0 statistics software (SPSS Inc, Chicago, IL)

## Results

### Bostrycin inhibited the proliferation of A549 cells

**Figure 1:**
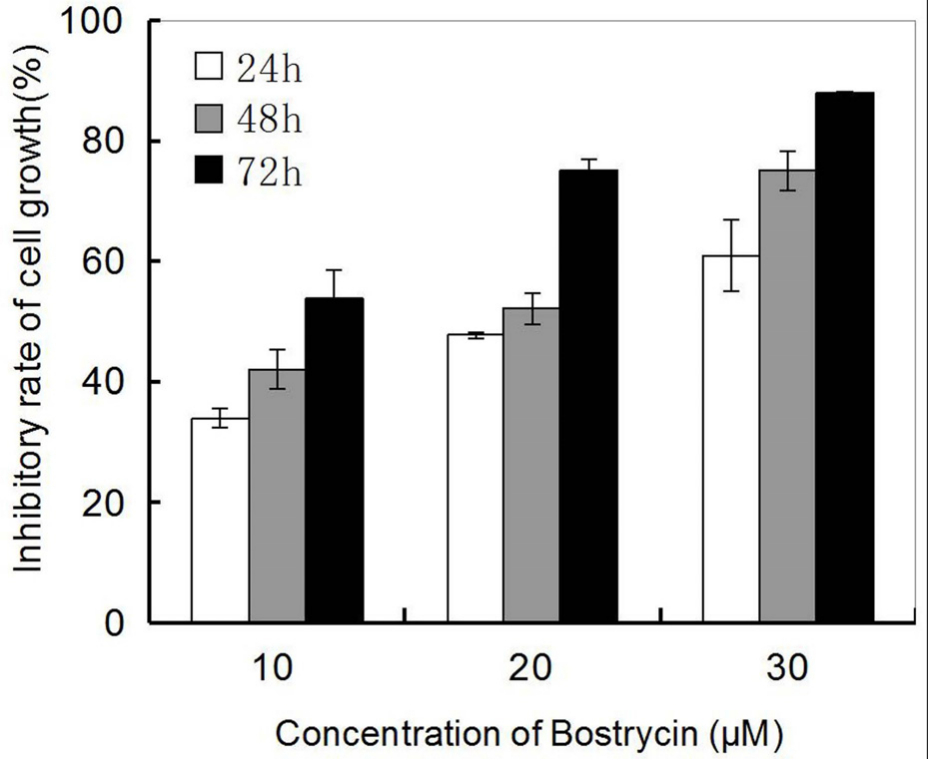
**Effect of Bostrycin on the proliferation of A549 cells by MTT assay**. A549 cells were treated with 10, 20, or 30 μM of bostrycin for 24 h, 48 h or 72 h. Negative control consisted of untreated cells, while the blank control was set up with only medium. Statistically significant differences were observed between groups treated with different bostrycin concentrations at each time point and between different time points at each concentration (all P < 0.05).

First, we used the MTT assay to detect effect of bostrycin on A549 cell proliferation. There was a dose-dependent and time-dependent inhibition of A549 cell proliferation by bostrycin (Figure 1) with an optimal linear relationship seen between 10-30 μΜ of bostrycin. This indicated that bostrycin could significantly inhibit A549 cell proliferation in vitro.

### Bostrycin induced cell cycle arrest and apoptosis in A549 cells

**Figure 2:**
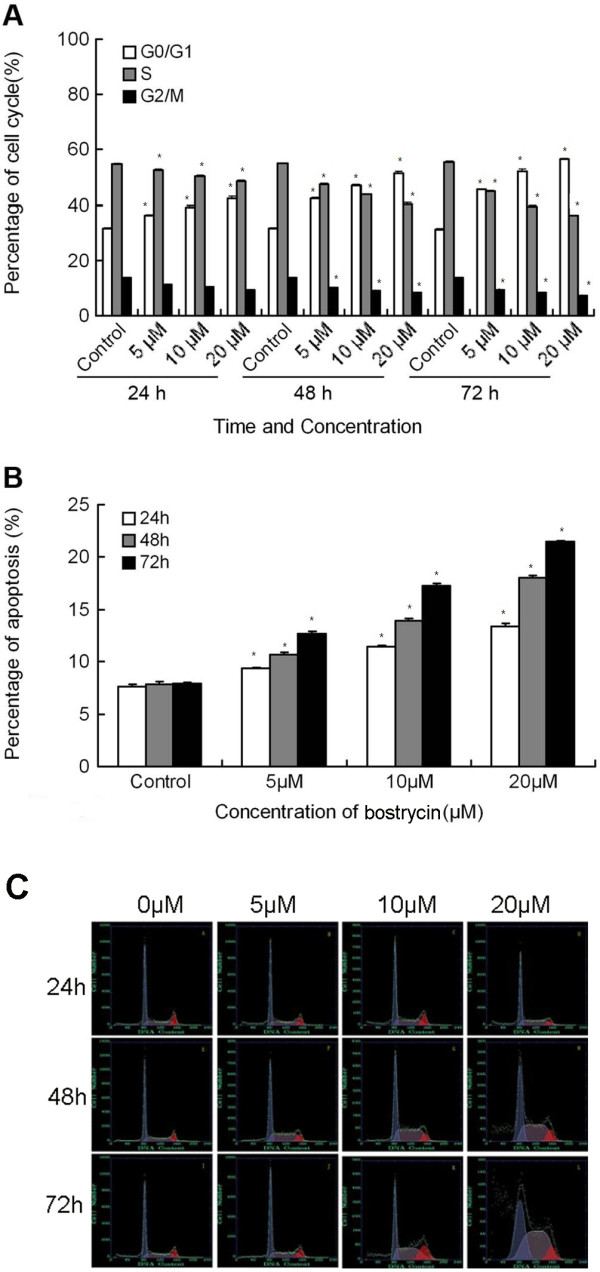
**Effect of Bostrycin on cell cycle and apoptosis detected by flow cytometry**. A549 cells were treated with 0, 5, 10 or 20 μM of bostrycin for 24 h, 48 h or 72 h. A) represents the percentage of A549 cells at different phases of the cell cycle at different time points and at different concentrations of bostrycin; B) represents the percentage of apoptotic A549 cells at different time points and at different concentrations of bostrycin; C) shows representative flow cytometry plots. *Indicates a statistically significant difference between the given group and its corresponding control group. Pair-wise multiple comparisons between groups were determined using Bonferroni's test with α = 0.017 adjustment.

Then, we used flow cytometry to determine cell cycle distribution and apoptosis in A549 cells exposed to different concentrations of bostrycin for 24, 48, and 72 hours. We showed a significant increase in the number of G_0_/G_1 _phase cells and a decrease in the number of S and G_2_/M phase cells after 72 hours of bostrycin treatment (Figure 2A). We also used propidium iodide staining to show that bostrycin induced apoptosis of A549 cells in a dose-dependent and time-dependent manner (Figure 2B). Figure 2C shows the flow cytometry data of cells treated with different concentrations of bostrycin for 24 h, 48 h and 72 h.

### Analysis of microRNA expression in A549 cells by microarrays and real-time RT-PCR

**Figure 3:**
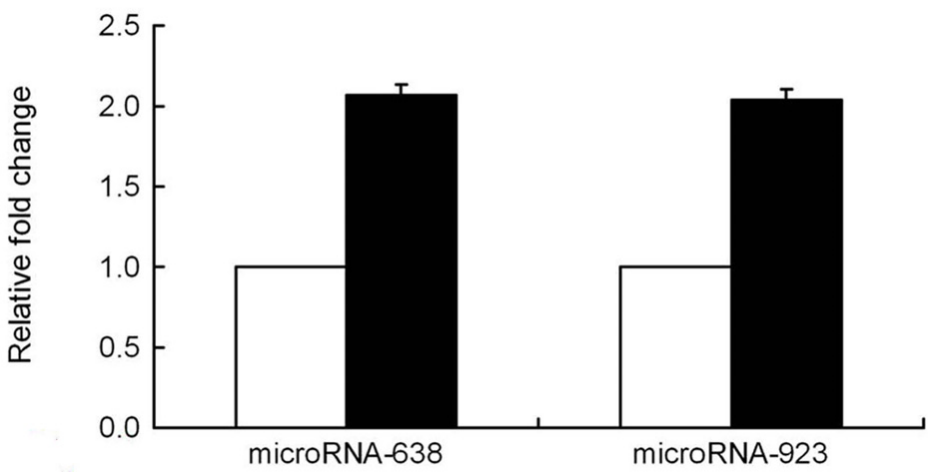
**Relative change in expression of microRNA-638 and microRNA-923 in A549 cells treated with bostrycin detected by microRNA real time PCR**. A549 cells were treated with 10 μM Bostrycin for 72 h and total RNA was isolated for microRNA real time PCR assay. Expression levels of microRNA-638 and microRNA-923 were determined as described. Untreated A549 cells were used as control. Each condition was repeated 4 times.

We used a gene chip probe techniques to detect changes in microRNA expression in bostrycin-treated A549 cells when compared with untreated cells. We found a statistically significant difference in the expression of fifty-four microRNAs (data not shown). We selected microRNA-638 and microRNA-923 for further validation with real-time RT-PCR since these two microRNAs showed the most significant difference. We used RT-PCR to demonstrate a significant upregulation in the levels of microRNA-638 and microRNA-923 in bostrycin-treated A549 cells. These data were consistent with our microarray analysis (Figure 3).

### Detection of p110α, p-Akt, and p27 levels in bostrycin-treated cells

**Figure 4:**
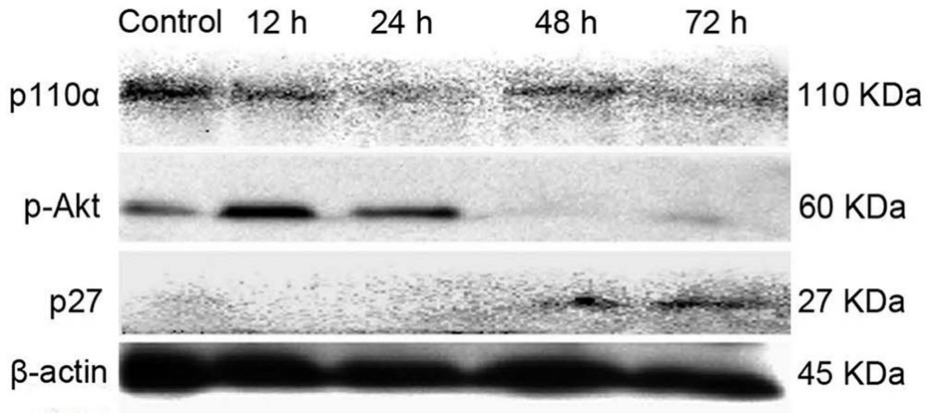
**Effects of Bostrycin on intracellular expression of p110α, p-Akt and p27 in A549 cells**. A549 cells were treated with 10 mol/L bostrycin for 12, 24, 48, or 72 hours. Cells were harvested, total proteins were extracted and immunoblotted for p110α, p-Akt and p27. Untreated A549 cells were used as a control. Beta-actin was used as loading control.

Finally, we detected the possible signal pathway involved in the effects of bostrycin on A549 cells. We showed by western blots that there was a decrease in the expression of p110α protein over time in bostrycin-treated A549 cells. Although there was an increase in the expression of p-Akt protein in cells treated with bostrycin for 12 hours, when compared with cells at the 0 hour time point, we showed a gradual decrease in p-Akt levels over time, with the most obvious reduction at 48 hours. We also showed a time-dependent increase in the levels of p27 protein in bostrycin-treated cells (Figure 4).

## Discussion

In this study, we demonstrated that bostrycin, a novel compound isolated from marine fungi in the South China Sea, inhibited cell proliferation, blocked cell cycle progression, and promoted apoptosis of lung cancer A549 cells. Our data also suggested that the PI3K/AKT signaling pathway may play a role in bostrycin-mediated inhibition of cell proliferation. Although bostrycin was previously shown to effectively inhibit cell growth and promote apoptosis in prostate cancer and gastric cancer [3,4], it has not been used in lung cancer cells. To our knowledge, ours is the first study demonstrating that bostrycin significantly inhibited the growth of A549 cells in a concentration- and time-dependent manner.

Regulation of the cell cycle and apoptosis is a major determinant dictating the development and progression of a number of cancers. PI3K/AKT inhibitors such as Tipifarnib, cause cell cycle arrest at the G_1 _or G_2_/M phase and induce apoptosis of human lung cancer cells [5,6] Our data were consistent with this study and showed that bostrycin treatment induced downregulation of PI3K/AKT signal pathway proteins, caused G_0_/G_1 _cell cycle arrest and promoted apoptosis in A549 cells.

PI3K is composed of a p110αsubunit and p85 subunit and the PI3K/AKT signaling pathway has been shown to play a role in the development and progression of lung cancer [7]. Increased Akt activity has been reported in the bronchial endothelial cells of long-term smokers [8,9] and persistently high levels of activated Akt was shown in bronchial endothelial cells from malignant tumors or precancerous lesions. Akt activation is thought to be related to poor prognosis of patients with lung cancer [10-12] and may be an important molecular target for treatment of lung cancer.

The PI3K/AKT signaling pathway inhibits apoptosis by inactivating important members of the apoptotic cascade, including caspase-9, forkhead, and proapoptotic Bad [13-15] and by upregulating the transcription and translation of antiapoptotic genes via NFκB [16] and cell cycle genes like cyclin D1 and p27 [17]. The p27 gene, a tumor suppressor, encodes a late G_1 _cyclin-dependent kinase inhibitor, whose activity is dependent on phosphorylation-dependent cytoplasmic translocation [18]. The PI3K/AKT pathway regulates p27 activity by 1) directly phosphorylating it at Thr159^, ^resulting in cytoplasmic translocation and inactivation of p27 or 2) phosphorylation and cytoplasmic translocation of AFX (a forkhead transcription factor), which downregulates p27 levels [19]. We used p110α expression levels as a marker of PI3K expression and showed a significant downregulation of p110α and p-Akt levels and an upregulation of p27 levels in bostrycin-treated A549 cells. These data suggest that p-Akt downregulation could inhibit cytoplasmic translocation of p27, causing a G_1 _cell cycle arrest of A549 cells. However, further studies are necessary to elucidate the mechanisms underlying bostrycin-mediated induction of apoptosis and attenuation of the PI3K/AKT signaling pathway in A549 cells. While we evaluated overall levels of phosphorylated Akt and p27 in this study, we would also like to detect changes in specific phosphorylation sites of these proteins, in order to more completely understand the mechanism of bostrycin action.

MicroRNAs are thought to play an important role in the development and progression of tumors [20]. Microarray analysis on 104 primary non-small cell lung carcinomas showed changes in the expression levels of 43 microRNAs in lung cancer tissue when compared with normal lung tissue [21]. Members of the let-7 family of microRNAs are known to inhibit growth of non-small cell lung carcinoma by inducing cell cycle arrest and apoptosis [22], while microRNA-126 inhibits the invasion of non-small cell lung carcinoma [23]. microRNA-25 and microRNA-205 have been used to predict survival and recurrence in lung cancer patients [24,25]. Exploring microRNA regulation may therefore provide useful information in developing new drug targets or identifying early disease markers [26]. MicroRNAs 638 and microRNA 923 were significantly upregulated in bostrycin-treated A549 cells. Both microRNAs might be related with tumor inhibition.

Interestingly, microRNAs have also been reported to play a regulatory role in the PI3K signaling pathway. Recombinant microRNA-126 was shown to downregulate the expression of p85β (a regulatory subunit of PI3K related to the stabilization and transmission of the PI3K signal) and p-Akt proteins in rectal cancer cells [27], and microRNA-7 inhibited the Akt pathway and reduced survival rates in spongiocytoma [28]. It is tempting to speculate that upregulation of microRNA-638 and microRNA-923 in bostrycin-treated A549 cells, accompanied by downregulation of the PI3K/AKT signaling pathway-associated proteins, p110α and p-Akt, are significantly related. We would like to dissect these pathways in greater detail in our upcoming studies, using luciferase assays to demonstrate direct targets of microRNA-638 and microRNA-923 in bostrycin-treated cells.

In conclusion, we demonstrated that bostrycin, a novel metabolite isolated from marine fungi, inhibited proliferation, blocked cell cycle progression and promoted apoptosis in pulmonary adenocarcinoma A549 cells. We also demonstrated 1) upregulation of tumor-suppressing transcriptional factors, the noncoding microRNA-638 and microRNA-923, and 2) downregulation of proteins associated with the PI3K/PI3K/AKT signaling pathway in bostrycin-treated cells, suggesting that bostrycin may be a new PI3K/AKT signal pathway-targeting drug for the treatment of pulmonary adenocarcinoma.

## Abbreviations

**PI3K**: Phosphoinositide 3-kinase; **AKt/PKB**: Protein Kinase B; **mAb**: monoclonal antibody; **IC50**: the half maximal inhibitory concentration.

## Conflict of interests

The authors declare that they have no competing interests.

## Authors' contributions

YBG: Conceived and designed the experiments;

WSC, JNH: Performed the experiments and analysed the data;

HLY, CMX, YCL, ZGS: Contributed reagents/material/analysis tools/.

All authors read an approved the final draft.

## Supplementary Material

Additional file 1 **Figure S1, Bostrycin (hydroxy-methoxy-tetrahydro-5-methyl anthracene dione)**. The file contains the molecular chemical structure of bostrycin. Click here for file
