# Supplementary figures and images for: Bostrycin inhibits proliferation of human lung carcinoma A549 cells via downregulation of the PI3K/Akt pathway
Source: J Exp Clin Cancer Res. 2011 Feb 8;30(1):17. doi: 10.1186/1756-9966-30-17 (PMC3041691; doi:10.1186/1756-9966-30-17)

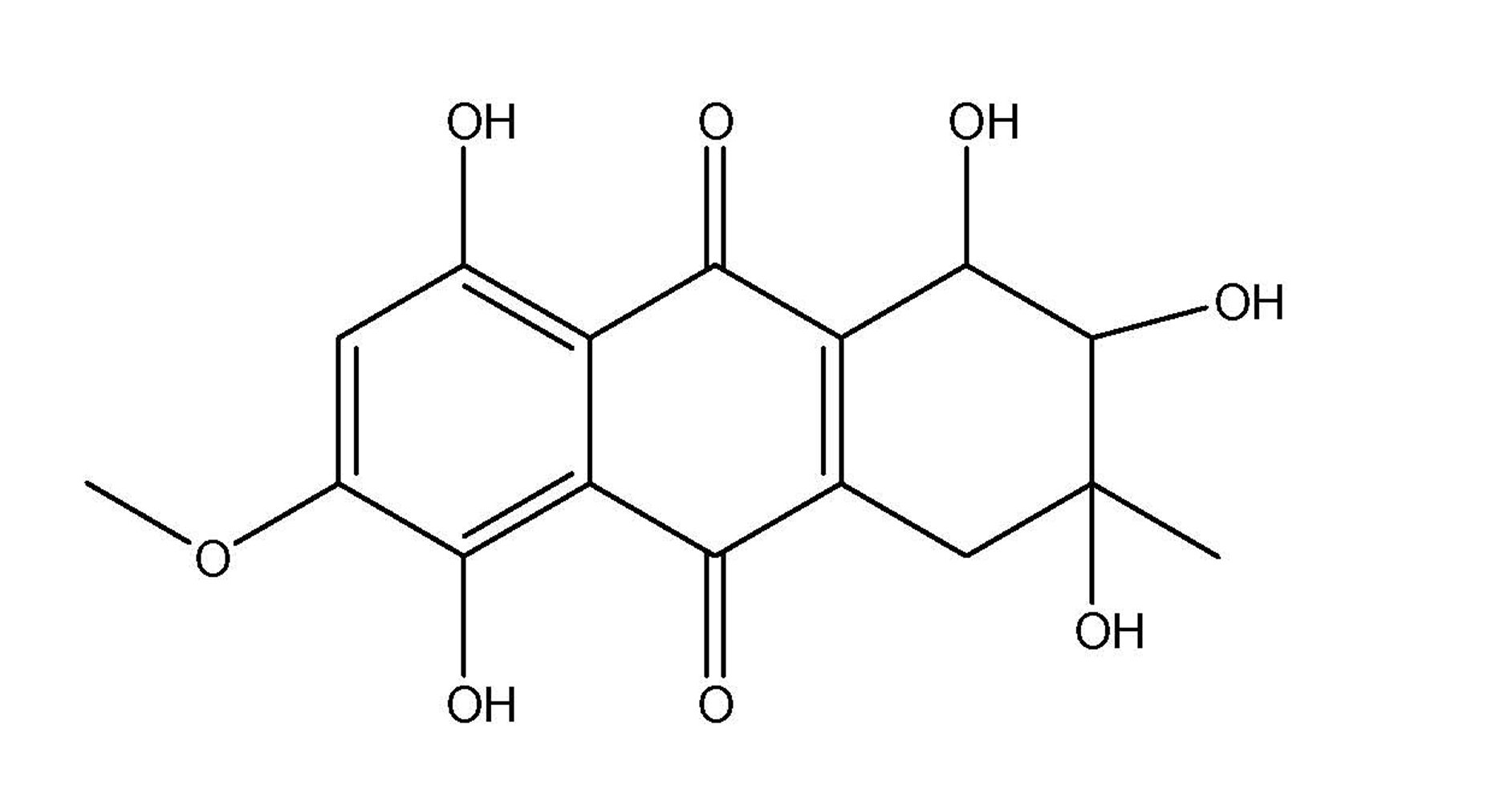

Supplement: Additional file 1 — Figure S1, Bostrycin (hydroxy-methoxy-tetrahydro-5-methyl anthracene dione). The file contains the molecular chemical structure of bostrycin. [file 1756-9966-30-17-S1.TIFF]
